# What predicts the unsuccess of bariatric surgery? An observational retrospective study

**DOI:** 10.1007/s40618-020-01398-z

**Published:** 2020-08-25

**Authors:** C. D’Eusebio, S. Boschetti, F. Rahimi, G. Fanni, A. De Francesco, M. Toppino, M. Morino, E. Ghigo, S. Bo

**Affiliations:** 1 Department of Medical Sciences, University of Turin, c.so AM Dogliotti 14, 10126 Turin, Italy; 2 Dietetic Unit, Città della Salute e della Scienza Hospital, Turin, Italy; 3 Department of Surgical Sciences, University of Turin, Turin, Italy

**Keywords:** Bariatric surgery, Percentage of excess weight loss, Predictors of weight loss, Retrospective observational study

## Abstract

**Purpose:**

Bariatric surgery (BS) has been recognized as an effective treatment for most patients with morbid obesity, but a variable range of patients failed to achieve a successful weight-loss. Controversial data are available about predictors of unsuccess. We aimed to retrospectively assess whether clinical baseline characteristics of patients submitted to sleeve gastrectomy (SL) or gastric bypass (GBP) were associated with unsuccessful weight-loss after 12 and 24-month follow-up.

**Methods:**

Three hundred patients who underwent BS from the 1st January 2016, with at least 24-months follow-up, were enrolled. Patients were divided according to their percentage of excess weight-loss (%EWL) either < 50% or ≥ 50% after 12 and 24-month follow-up.

**Results:**

None of the patients was lost at follow-up; 56 (18.7%) patients showed a %EWL < 50% at 24 months. Age, neck circumference, obstructive sleep apnea (OSA) were significantly higher, while total cholesterol and %EWL 6-months lower in those with %EWL < 50% at 12-months. Age, neck circumference, male and OSA rates were increased, while %EWL at 6-months lower in patients with %EWL < 50% at 24-months. In a multiple regression model, age (OR = 1.076; 95% CI 1.029–1.125; *p* = 0.001; OR = 1.066; 1.027–1.107; *p* < 0.001) and %EWL at 6-months (OR = 0.876; 0.840–0.913; *p* < 0.001; OR = 0.950; 0.928–0.972; *p* < 0.001) were associated with %EWL < 50% both at 12- and 24-months, respectively, and neck circumference (OR = 1.142; 1.011–1.289; *p* = 0.032) with %EWL < 50% at 24-months.

**Conclusion:**

Older age, larger neck circumference, and %EWL at 6-months were significantly associated with BS unsuccess, showing almost 90% of those patients an unsuccessful weight-loss early after surgery. Further larger studies with longer follow-up are needed to confirm these results.

## Introduction

Bariatric surgery (BS) has recognized as an effective treatment for patients with morbid obesity, i.e. for those individuals with a body mass index (BMI) ≥ 40 kg/m^2^ or a BMI ≥ 35 kg/m^2^ with comorbidities [1]. The definition of BS success is an important issue. Different studies referred to the minimum weight/BMI achieved after surgery [2] and to the weight loss either as a percentage of total body weight (%TWL) [3, 4], or as a percentage of the weight in excess (%EWL) [3, 5], or as a percentage of the excess of BMI lost (%EBMIL) [6].

Percentage of excess weight loss (%EWL) is one of the most accepted criteria. It is calculated as the difference between baseline weight and weight after BS divided for the difference between baseline weight and the ideal weight, and it is expressed as a percentage. The %EWL cutoff ≥ 50% has been proven to be a specific and sensitive criterion for BS success [7]. The mean %EWL values reported at 10-years after surgery were 45.9, 56.7, 58.3 and 74.1% for laparoscopic adjustable gastric banding (LAGB), gastric bypass (GBP), sleeve gastrectomy (SG) and biliopancreatic diversion, respectively [8].

Several baseline patient characteristics, such as increased fasting glucose levels [6, 9], lower scores of the Homeostasis Model Assessment-Insulin Resistance (HOMA-IR) index [10], and increased waist circumference values [11], have been associated with poor weight loss. Moreover, early weight loss trajectories, i.e. weight loss trends in the period immediately following BS, has been demonstrated to predict subsequent weight reduction [12]. On the other hand, associations between pre-bariatric BMI and weight loss after BS have been shown highly contrasting, since either inverse [3, 13, 14], direct [4, 15] or no [16–19] relationships have been reported. Similarly, sex and age have been correlated with weight loss in some studies [4], but not in others [20] and the presence of personality disorders, psychiatric diseases, eating disorders or stressful life events (such as binge eating, sweet eating, depression, anxiety, low self-esteem, alcohol abuse, previous sexual abuse) have resulted both predictive [21] and not predictive [22] of poor BS success. Furthermore, studies presented great heterogeneity with respect to follow-up durations (from 6 months [3] to 7 years [4]), sample size (from 20 patients [18] to about 1300 [20]), type of surgery (intragastric balloon (IGB) [3], LAGB [19], GBP [6], SG [4] or biliopancreatic diversion [23]), and age and ethnic characteristics of participants [2, 4, 19].

Determining the causes of BS unsuccess is a priority to optimize access to this procedure and therefore sanitary resources. The aim of this retrospective observational study was to assess whether clinical and/or anthropometric baseline characteristics of patients who underwent GBP or SG were associated with poor %EWL after 12 and 24-months of follow-up.

## Patients and methods

This was a retrospective observational study.

We collected data from the clinical records of the first 300 patients who underwent BS at the General Surgery Department of the “Città della Salute e della Scienza” Hospital of Turin, University of Turin, from the 1st January 2016 and meeting the below-reported criteria.

Inclusion criteria were: (1) age from 18 to 65 years old; (2) BMI ≥ 35 kg/m^2^ with comorbidities or BMI ≥ 40 kg/m^2^, with numerous unsuccessful attempts to lose weight; (3) a minimum 2-year follow-up at our Obesity Unit. Exclusion criteria were: (1) secondary causes of obesity (e.g. hypothalamic diseases, endocrine diseases); (2) patients unable to participate in prolonged medical follow-up; (3) associated comorbidities or diseases impacting on weight loss (for example, patients who performed BS in prevision of a transplantation). Data were collected from the clinical records of all the patients, since they performed different post-BS visits (i.e. surgical, nutritionist visits).

### Ethical aspects

Patients gave their informed consent to the processing of their data. The study was approved by the local Ethics Committee and was in accordance with the Declaration of Helsinki principles.

### Pre-operative management

All patients first underwent a clinical examination carried out by a dietician and a MD with specific expertise in management of patients undergoing BS. During the visit, data relative to eating behaviors, previous attempts of weight loss and presence of comorbidity were collected. All patients received verbal and written dietary, exercise and behavioral recommendations. To obtain weight-loss before intervention, in order to reduce operative risks, an individually prescribed diet was given in accordance with the Mediterranean diet composition (45–55% carbohydrates, < 10% sugars, 30% fats, < 10% saturated fats, 15–25% proteins, 20–30 g fiber) with an energy restriction ranging from 500 to 1000 kcal and based on the individual caloric requirements and usual intake.

The following anthropometric measurements were assessed in all patients: weight, height, waist circumference, and neck circumference. A fasting blood sample was drawn from all patients and glucose and lipid values were centrally measured.

The preoperative evaluation consisted in clinical examinations carried out by a surgeon, an anesthetist, and a psychiatrist, with expertise in the management of BS patients. All patients received information about the post-BS diet before undergoing surgery.

### Bariatric surgery

In our hospital, either SG or GBP were performed. Both techniques were totally carried out by laparoscopic approach.

The type of surgery was based on the following patient features: BMI, age, gender, body fat distribution, presence of type 2 diabetes mellitus (T2DM), hiatal hernia, gastroesophageal reflux disease, patient’s expectations/realistic goals, long-term treatment for a coexisting disease or condition for which absorption and pharmacokinetics of drugs are of major concern [1].

GBP is expected to have a greater effect on metabolic improvement, weight loss and maintenance with respect to SG. On the other hand, this latter is preferable to GBP for the lower surgical complexity and less frequent long-term nutritional and surgical complications [1].

### Sleeve gastrectomy

First, a dissection by Ligasure™ of the gastrocolic ligament along the greater curve was done (from 5 cm proximal to pylorus up to the gastro-esophageal junction). A 32F bougie was placed trans-orally until the pylorus as a guide for resection. Then, a resection of the left-lateral part of the stomach, including the complete fundus, was performed, from the antrum to the angle of His, by multiple firing of a linear cutter stapler EndoGIA (Medtronic, USA). Healing of the staple line was tested intraoperatively by methylene blue solution introduced by a nasogastric tube. The resected stomach was finally removed through a trocar site.

### Gastric by-pass

GBP was performed with robotic technique (Da Vinci™ Surgical System, Intuitive, USA) in the majority of cases. A 30 mL gastric pouch was performed, thus separating it from the excluded stomach by a linear cutter stapler; then, an ante-colic ante-gastric 150-cm Roux-en-Y limb and a 100 cm biliopancreatic limb were carried out in all cases. In the robotic technique, the double loop technique was adopted, with a double layer end-to-side handsewn gastro-jejunal anastomosis and mechanical or handsewn side-to-side jejuno-jejunal anastomosis, finally obtaining the Roux-en-Y limb. In the laparoscopic technique, the gastro-jejunal anastomosis was performed with a circular stapler and the jejuno-jejunal anastomosis with a linear stapler. A methylene blue test was always performed.

### Post-operative management

Postoperatively, patients were advised to follow a 7-meals liquid diet during the first month after surgery, then a 5-meals soft/smooth diet was prescribed for the following four months. Starting from the sixth month onwards, foods with normal consistence were allowed and caloric sources were distributed as follows: proteins 22%, lipids 28%, carbohydrates 50%. The caloric amount prescribed was based on the Harris-Benedict formula, considering an energy restriction ranging from 500 to 1000 kcal.

Patients were followed-up at our obesity unit at 1, 2, 6, 12 and 24 months after surgery. Daily vitamin supplementation was prescribed for the first year after surgery; thereafter, supplementation was individualized according to specific needs.

During each visit, patients were weighted, dietary habits were assessed, and the %EWL was calculated as:

$$ \left[ {\left( {{\text{pre-BS weight }} - {\text{ weight at the time of visit}}} \right)/\left( {{\text{pre -BS weight }} - {\text{ ideal weight}}} \right)} \right]\, \times \,{1}00. $$

Weight corresponding to the BMI = 25 kg/m^2^ was considered as the ideal body weight. An unsuccessful weight loss after BS was defined as %EWL < 50%, according to guidelines [1].

### Measurements

Weight was measured with patient wearing light clothes and no shoes to the nearest 0.1 kg by a digital scale with a capacity of 300 kg (Wunder Sa.Bi.srl). Height was measured to the nearest 0.1 cm with a Stadiometer SECA 220 measuring rod (Hamburg, Germany). Waist and neck circumferences were assessed by a plastic tape meter at the umbilicus level and under the cricoid cartilage, respectively.

T2DM and arterial hypertension were diagnosed by MDs in accordance with guidelines. Obstructive sleep apnea (OSA) was hypothesized in the presence of excessive daytime sleepiness, snoring, and choking or gasping during sleep, enlarged neck circumference, and an intermediate to high risk score at the STOP-Bang questionnaire [24]. The diagnosis was confirmed by a sleep-expert neurologist by means of further exams (in-laboratory polysomnography, unattended home sleep apnea testing, etc.).

### Statistical analyses

Variables are presented as mean ± standard deviation (SD). Patients were divided according to their %EWL either < 50 or ≥ 50% after 12 and 24-months of follow-up.

A univariate logistic analysis was performed to test the variables associated (crude ORs) with unsuccessful weight loss at 12- and 24-months after BS (dependent variables). Then, the variables that were significantly associated with %EWL < 50 at 12- and 24-months were inserted in a multiple logistic regression model (adjusted ORs). The Hosmer–Lemeshow test was used to evaluate the goodness-of-fit of the multivariate models.

## Results

The characteristics of the participants are reported in Table 1. The majority were women and the most common surgical procedure was sleeve gastrectomy. There was a high rate of current smokers; all patients were required to quit smoking permanently before BS. None of the patients was lost at follow-up.

**Table 1:** Baseline characteristics of the whole sample

Number	300
Age (years)	44.6 ± 10.3
Males (%)	10.6
Smoking habits (%)	
Current smokers	57.0
Past smokers	20.3
Never smoked	22.7
Job (%)	
Unemployed/housewife/retired	38.3
Employee	40.3
Manual worker	19.0
Other	2.3
Sleeve gastrectomy (%)	77.7
Gastric bypass (%)	22.3
Pre-bariatric weight (kg)	118.2 ± 22.9
Height (cm)	163.0 ± 9.0
Pre-bariatric BMI (kg/m^2^)	44.4 ± 8.0
Waist circumference (cm)	123.4 ± 15.4
Waist-to-height ratio	0.76 ± 0.09
Neck circumference (cm)	38.8 ± 3.6
OSA (%)	21.7
Fasting glucose (mg/dL)	107.7 ± 29.3
Type 2 diabetes mellitus (%)	22.7
Hypoglycemic drugs (%)^a^	44.4
Insulin (%)^a^	11.1
Arterial hypertension (%)	34.7
Statins (%)	12.7
Total cholesterol (mg/dL)	196.5 ± 37.8
HDL-cholesterol (mg/dL)	47.2 ± 13.0
Triglycerides (mg/dL)	146.3 ± 63.1

*BMI* body mass index, *OSA* obstructive sleep apnea

^a^Percentage of patients with type 2 diabetes treated with the drug

In the first month after BS, mean caloric intake was 986 ± 124 kcal (21% proteins, 33% fats, 46% carbohydrates). The percentages of patients with unsuccessful weight loss, defined as %EWL < 50%, progressively declined from 2 to 12 months, being 97, 41, 17% at 2, 6, 12 months after BS, respectively. At 24 months, the prevalence of %EWL < 50% did not further reduce (19%). Median (mean ± SD) values of %EWL were 22.9 (24.0 ± 18.8), 54.4 (54.8 ± 33.0) and 71.2 (68.9 ± 55.1) at 2, 6 and 12 months, respectively. At 24 months, the prevalence of %EWL did not further reduce, being median (mean ± SD) values of %EWL 73.3 (72.6 ± 38.8).

Patients were divided according to their %EWL (either lower or higher 50%) at 12 months (Table 2) and at 24 months (Table 3) after surgery. Age, neck circumference, rate of OSA were significantly higher, while total cholesterol and %EWL at 6-months lower in those with unsuccessful weight loss at 12 months. Indeed, patients with unsuccessful weight loss at 12-months were more frequently on statins (Table 2).

**Table 2:** Baseline characteristics by %EWL at 12 months (left) and their association (right) with %EWL < 50% at 12 months

	%EWL ≥ 50%	%EWL < 50%	OR	95% CI	*p*
Number	248	52			
Age (years)	43.4 ± 10.3	50.1 ± 8.6	1.076	1.046–1.109	< 0.001
Males (%)	9.3	17.3	2.047	0.984–4.052	0.09
Smoking habits (%)					
Current smokers	56.9	57.7	1^b^		
Past smokers	21.4	15.4	0.957	0.856–1.069	0.43
Never smoked	21.8	26.9	1.031	0.927–1.147	0.58
Job (%)					
Unemployed/housewife/retired	37.9	40.4	1^b^		
Employee	40.7	38.5	0.982	0.892–1.082	0.73
Manual worker	19.8	15.4	0.959	0.850–1.081	0.49
Other	1.6	5.8	1.278	0.958–1.707	0.10
SG (%)	79.8	67.3	0.520	0.302–0.913	0.06
Pre-bariatric weight (kg)	118.9 ± 21.8	114.6 ± 27.6	0.919	0.979–1.003	0.22
Height (cm)	163.2 ± 8.6	162.4 ± 10.0	0.991	0.962–1.019	0.59
Pre-bariatric BMI (kg/m^2^)	44.7 ± 7.9	43.1 ± 8.5	0.972	0.937–1.011	0.18
% pre-bariatric weight loss	− 0.88 ± 3.7	− 0.41 ± 4.5	1.033	0.954–1.118	0.42
Waist circumference (cm)	123.3 ± 14.6	124.3 ± 19.1	1.010	0.988–1.022	0.65
Neck circumference (cm)	38.6 ± 3.4	39.9 ± 4.4	1.097	1.027–1.171	0.021
OSA (%)	17.7	40.4	3.141	1.823–5.378	< 0.001
Fasting glucose (mg/dL)	107.4 ± 28.4	109.3 ± 33.3	1.002	0.993–1.010	0.65
Type 2 diabetes mellitus (%)	23.0	21.2	0.899	0.474–1.622	0.77
Hypoglycemic drugs (%)^a^	47.4	54.6			
Insulin (%)^a^	12.3	9.1			
Arterial hypertension (%)	34.7	34.6	0.997	0.582–1.6779	0.99
Statins (%)	11.7	17.3	1.581	0.696–3.587	0.27
Total cholesterol (mg/dL)	198.9 ± 36.9	184.6 ± 40.1	0.989	0.981–0.998	0.014
HDL-cholesterol (mg/dL)	46.8 ± 12.9	49.0 ± 13.2	1.13	0.994–1.031	0.26
Triglycerides (mg/dL)	146.2 ± 66.0	146.7 ± 47.8	1.000	0.996–1.004	0.96
%EWL at 6-month	60.9 ± 20.4	25.5 ± 57.4	0.893	0.867–0.917	< 0.001

*%EWL* percentage excess weight loss, *SG* sleeve gastrectomy, *BMI* body mass index, *OSA* obstructive sleep apnea

^a^Percentage of patients with type 2 diabetes treated with the drug

^b^Dummy variables

**Table 3:** Baseline characteristics by %EWL at 24 months (left) and their association (right) with %EWL < 50% at 24 months

	%EWL ≥ 50%	%EWL < 50%	OR	95% CI	*p*
Number	244	56			
Age (years)	43.4 ± 10.1	49.9 ± 9.7	1.074	1.045–1.106	< 0.001
Males (%)	8.2	21.4	3.054	1.557–5.860	0.005
Smoking habits (%)					
Current smokers	57.4	55.4	1^b^		
Past smokers	20.1	21.4	1.015	0.906–1.139	0.79
Never smoked	22.5	23.2	1.010	0.905–1.127	0.86
Job (%)					
Unemployed/housewife/retired	38.1	39.3	1^b^		
Employee	40.6	39.3	0.991	0.896–1.094	0.85
Manual worker	19.7	16.1	0.967	0.854–1.094	0.59
Other	1.6	5.4	1.268	0.941–1.707	0.12
SG (%)	77.9	76.8	0.940	0.535–1.714	0.86
Pre-bariatric weight (kg)	118.3 ± 22.4	117.8 ± 25.4	0.999	0.988–1.010	0.89
Height (cm)	162.9 ± 8.7	163.9 ± 9.5	1.013	0.985–1.041	0.44
Pre-bariatric BMI (kg/m^2^)	44.6 ± 8.1	43.6 ± 7.7	0.984	0.951–1.015	0.41
% pre-bariatric weight loss	− 0.78 ± 3.9	− 0.87 ± 3.5	0.994	0.921–1.072	0.88
Waist circumference (cm)	123.0 ± 14.6	125.3 ± 18.6	1.010	0.994–1.026	0.33
Neck circumference (cm)	38.5 ± 3.3	40.4 ± 4.4	1.146	1.074–1.225	< 0.001
OSA (%)	18.9	33.9	2.210	1.281–3.761	0.015
Fasting glucose (mg/dL)	106.7 ± 28.8	112.2 ± 31.2	1.006	0.998–1.014	0.20
Type 2 diabetes mellitus (%)	22.1	25.0	1.173	0.653–2.041	0.64
Hypoglycemic drugs (%)^a^	48.2	50.0			
Insulin (%)^a^	11.1	14.3			
Arterial hypertension (%)	34.4	35.7	1.058	0.630–1.751	0.85
Statins (%)	13.1	10.7	0.795	0.314–2.011	0.63
Total cholesterol (mg/dL)	198.3 ± 37.7	188.3 ± 37.6	0.993	0.986–0.999	0.07
HDL-cholesterol (mg/dL)	47.4 ± 13.1	46.5 ± 12.3	0.994	0.975–1.013	0.63
Triglycerides (mg/dL)	146.4 ± 65.8	145.9 ± 50.3	1.000	0.996–1.004	0.96
%EWL at 6-month	59.9 ± 25.9	32.5 ± 48.2	0.945	0.928–0.962	< 0.001

*%EWL* percentage excess weight loss, *SG* sleeve gastrectomy, *BMI* body mass index, *OSA* obstructive sleep apnea

^a^Percentage of patients with type 2 diabetes treated with the drug

^b^Dummy variables

Age, neck circumference, male sex, and rate of OSA were increased, while %EWL at 6-months lower in patients with unsuccessful weight loss at 24 months. The rate of EWL < 50% was very high at 2 months in both groups (96 and 100%), as expected, but declined at 6-months (31 and 88%, in patients with successful and unsuccessful weight loss at 24 months, respectively).

Patients with successful weight loss at 24-months showed also significantly lower fasting values at 24-months of glucose (81.7 ± 12.8 vs 95.9 ± 14.7 mg/dL, *p* < 0.001; median change − 20 vs − 6 mg/dL), triglycerides (83.6 ± 35.9 vs 124.9 ± 64.9 mg/dL, *p* < 0.001; median change − 57 vs − 16.5 mg/dL), and higher values of HDL cholesterol (55.8 ± 15.1 vs 51.1 ± 12.5 mg/dL, *p* = 0.03; median change + 4 vs + 1 mg/dL). Total cholesterol values were reduced, but the difference between patients with successful and unsuccessful weight loss at 24-months was not significant (186.0 ± 32.6 vs 192.2 ± 36.8 mg/dL, *p* = 0.21; median change − 12.5 vs + 4 mg/dL). No patients discontinued statin therapy, while insulin was discontinued in all patients and hypoglycemic drugs in all except 5 patients (1 in the successful and 4 in the unsuccessful groups, respectively).

In a multiple regression model, age and %EWL at 6 months remained significantly associated with unsuccessful weight loss both at 12 months and at 24 months, and larger neck circumference with unsuccess at 24-months (Table 4). Risk of BS unsuccess almost doubles with every decade of age (OR = 1.82; 95% CI 1.27–2.59, *p* < 0.001).

**Table 4:** Association between baseline variables and %EWL < 50% at 12-months (upper) and at 24-months (lower) in a multiple logistic regression model

%EWL < 50% at 12-months	OR	95% CI	*p*
Age (years)	1.076	1.029–1.125	0.001
Neck circumference (cm)	1.039	0.930–1.160	0.50
Total cholesterol (mg/dl)	0.993	0.983–1.003	0.18
OSA	1.793	0.692–4.651	0.23
%EWL at 6-month	0.876	0.840–0.913	< 0.001

%EWL < 50% at 24-months	OR	95% CI	*p*
Age (years)	1.066	1.027–1.107	< 0.001
Male sex	1.145	0.329–3.989	0.83
Neck circumference (cm)	1.142	1.011–1.289	0.032
OSA	0.665	0.281–1.575	0.35
%EWL at 6-month	0.950	0.928–0.972	< 0.001

Goodness-of-fit by the Hosmer–Lemeshow test *p* = 0.99 (for %EWL < 50 at 12-months), and *p* = 0.69 (for %EWL < 50 at 24-months)

*%EWL* percentage excess weight loss, *OSA* obstructive sleep apnea

## Discussion

Older age and %EWL at 6 months were significantly associated with BS unsuccess both after 12 and 24 months, and larger neck circumference with unsuccess at 24-months after BS. Almost 90% of patients with unsuccessful weight-loss at 24 months had already showed an unsuccessful weight-loss at 6 months. These results suggested that patients at risk of BS failure could be detected as early as 6 months after surgery.

The role of age in influencing post-BS weight loss is still controversial. Older age has been associated with lower weight loss at 6 months [3, 4, 11], 12 months [4, 6, 17, 19] and 24 months [4] after surgery by many, but not all studies [9, 15, 16, 18, 20–22]. We found a strong direct relationship between age and BS unsuccess at 24 months: the risk of unsuccess almost doubled with every increase in decade of age. Differences among studies might be due to differences in mean ages of cohorts, ranging from 34 [10] to 45 years [21], ethnicity [4, 17, 19], and employed surgical techniques. The reduced weight loss might be explained by decreased age-related resting energy expenditure (REE) due to: reduction in fat-free mass [25]; presence of adaptive thermogenic processes [26] or lower capacity to metabolize substrates [27]; the increased prevalence of comorbidity [28]; the reduction in exercise [29] and lower compliance to the received lifestyle recommendations [20].

Among our patients, the most powerful predictor of weight loss both at 12 and 24 months was %EWL at 6 months. Several studies investigated the relationships between early weight loss and later weight outcomes, even if the definition of satisfactory early %EWL varies greatly. After RYGB, the EWL velocity (calculated as the difference between %EWL at 3 and %EWL at 1 months divided by 10 weeks) was an independent predictor for %EWL at 36 months [29]. A strong correlation between %EWL at 1 month and %EWL at 1 year and 2 years was found [30]. Patients who lost < 30% of their initial excess weight at 6 months are unlikely to lose more than 50% at 24 months [31]. Patients in the lowest group of %EWL at 1 and 3 years after BS already showed a lower mean %EWL at 1 month [32]. A value of %EWL > 40% at 6 months after BS predicted weight loss at 2 years [33]. Patients compliance to dietary/exercise recommendations and scheduled follow-up shortly after BS was probably one of the most important factors linked to early %EWL, as demonstrated by several researchers [34–36]. Meanwhile, individual responses to surgery due to genetic [36], hormonal [37–39] and psychosocial factors [40–42] might play an important role in determining successful weight loss after BS.

Anatomical and absorption BS-induced modifications are not the only determinant of weight loss. The gastrointestinal tract releases several polypeptide hormones acting on the hunger/satiety pattern and on energy balance. SG-induced weight loss is at least in part linked to a reduction in the release of the orexigenic ghrelin, due to the removal of the fundus and a part of the body of the stomach [37], and inter-individual differences in ghrelin release might account for differences in post-BS weight loss. After an early reduction in ghrelin, a late compensatory raise in ghrelin occurs in response to weight reduction following BS [43]. This finding could explain the lower weight decrease seen after SG in some cohorts [44]. GBP-induced weight-loss is linked with raised secretion of glucagon-like peptide-1 (GLP-1) and peptide YY (PYY), subsequent to the quick reaching of food bolus to distal jejunum [45] with an overall suppression of hunger [38]. Among our patients, no association between type of BS (SG or GBP) and weight loss was found. Indeed, the choice of surgical intervention was linked to the expected average impact on weight loss (higher for GBP) and the patient specific anatomical and clinical characteristics. This could have influenced the described associations.

We found an increased baseline neck circumference, a predictor of OSA and metabolic syndrome, in subjects with unsuccessful weight loss at 24 months. The association remained significant in the multivariate model, after adjusting for gender and presence of OSA, suggesting the potential of a simple and cost-saving measurement, which is less affected by the changes in the body size occurring during the course of the life. This result is intriguing, considering the emerging role of neck circumference in the prediction of major outcomes such as cardiovascular diseases and mortality [46, 47].

We failed to find correlations between preoperative BMI/weight and weight loss after surgery, in line with some studies [16–19], but contrarily to others reporting a negative association between baseline BMI, waist circumference, waist-to-height ratio and post-BS weight loss at different follow-up times [3, 6, 9, 11, 15, 17, 20, 29]. Indeed, weight and BMI are both inaccurate measures of body composition with respect to fat distribution [48].

In conclusion, patients with a lower %EWL at 6 months after surgery might be at higher risk of future unsuccess. These individuals, above all those with a larger neck circumference at baseline, might require an aggressive lifestyle intervention with more frequently scheduled follow-up examinations. Moreover, older patients might be worth receiving personalized indications, even by taking into account their preferences, with the aim to achieve a higher compliance.

The present study has many limitations: the short follow-up, the lack of socio-economic data, physical activity level, body fat measurements and hunger peptides blood concentrations. The retrospective observational design of the study does not allow to control for other confounders. The majority of our patients were women; however, the same gender distribution was reported by most published studies on patients submitted to bariatric surgery as well [4, 6, 9, 11–13, 15–17, 19–23, 29, 31, 33, 35, 36, 38, 40, 42, 48]. The number of the analyzed patients was low, but the post-hoc power analysis found that the study had a 98.5% power (with *α* = 0.05) to detect early weight-loss differences between the two groups (unsuccessful *vs* successful BS). We have chosen %EWL < 50% as an index of BS unsuccess. Indeed, the %EWL < 50% cutoff proved to be clinically relevant because it was able to discriminate also the metabolic response to BS in our patients, in line with literature that referred to this index to standardize outcomes of metabolic surgery [49, 50].

Further, larger studies with longer follow-up are needed to confirm our results in order to obtain simple prognostic factors that might improve the clinical practice by identifying individualized approaches to post-BS patients with different intensity based on the individual risk of failure.
